# Availability of healthier vs. less healthy food and food choice: an online experiment

**DOI:** 10.1186/s12889-018-6112-3

**Published:** 2018-11-29

**Authors:** Rachel Pechey, Theresa M. Marteau

**Affiliations:** Behaviour and Health Research Unit, Institute of Public Health, University of Cambridge, Forvie Site, Cambridge, CB2 0SR UK

**Keywords:** Food, Healthiness, Availability, Cognitive load, Socioeconomic status

## Abstract

**Background:**

Our environments shape our behaviour, but little research has addressed whether healthier cues have a similar impact to less healthy ones. This online study examined the impact on food choices of the number of (i) healthier and (ii) less healthy snack foods available, and possible moderation by cognitive load and socioeconomic status.

**Methods:**

UK adults (*n* = 1509) were randomly allocated to one of six groups (two cognitive load x three availability conditions). Participants memorised a 7-digit number (7777777: low cognitive load; 8529713: high cognitive load). While remembering this number, participants chose the food they would most like to eat from: (a) two healthier and two less healthy foods, (b) six healthier and two less healthy foods, or (c) two healthier and six less healthy foods.

**Results:**

Compared to being offered two healthier and two less healthy options, the odds of choosing a healthier option were twice as high (Odds Ratio (OR): 2.0, 95%CI: 1.6, 2.6) with four additional healthier options, while the odds of choosing a less healthy option were four times higher (OR: 4.3, 95%CI: 3.1, 6.0) with four additional less healthy options. There were no significant main effects or interactions with cognitive load or socioeconomic status.

**Conclusions:**

This study provides a novel test of the impact of healthier vs. less healthy food cues on food choice, suggesting that less healthy food cues have a larger effect than healthier ones. Consequently, removing less healthy as opposed to adding healthier food options could have greater impact on healthier choices. Studies are now needed in which choices are made between physically-present foods.

**Electronic supplementary material:**

The online version of this article (10.1186/s12889-018-6112-3) contains supplementary material, which is available to authorized users.

## Background

Non-communicable diseases (NCDs), including diabetes, cardiovascular disease and cancer, now cause the majority of premature preventable deaths worldwide [1, 2]. Patterns of unhealthy behaviour, including excessive energy intake, are key contributors to these NCDs, and are socially patterned, i.e. less healthy behaviours are generally more common amongst the poorest, contributing in turn to the substantial socioeconomic inequalities in life expectancy and years lived in good health.

One strategy that may be effective in targeting these behavioural risk factors is to target the physical micro-environment, addressing the multiple cues – aspects of our environments that can influence behaviour – which act detrimentally by limiting healthier options or promoting less healthy ones [3]. This approach (sometimes termed ‘choice architecture’ or ‘nudging’) [4–6] is based on dual process models of behaviour [7, 8]. It has been hypothesised that interventions targeting non-conscious processes regulating behaviour are more effective than more information-based interventions, as they do not necessarily rely on individuals’ cognitive resources [3, 9]. One such environmental cue is the availability (including both the number and range) of healthier vs. less healthy foods, which represents one of the top three interventions suggested in the McKinsey Global Institute report on obesity [10] as having the highest likely impact across the population. While the mechanisms underlying the effects of altering availability have not been explored to our knowledge, increasing the availability of product(s) may influence consumption by increasing the visibility or salience of these products to consumers, and/or increased options may lead to these appealing to a wider range of people. Evidence is beginning to accumulate to support the effectiveness of targeting product availability to change behaviour [11–14].

One choice when designing interventions to alter availability is whether to increase healthier foods, decrease less healthy foods or both simultaneously. Thus far, there is a paucity of evidence on this, although observational data suggests that the availability of less healthy foods but not fruit and vegetables is associated with body mass index (BMI) [15]. Establishing if there is a difference in response to healthier vs. less healthy food cues could help prioritise interventions that are likely to be most effective to change behaviour.

Looking at food cues beyond product availability, evidence comparing responses to healthier vs. less healthy food cues remains limited. There are a small number of observational studies demonstrating that individuals may be more responsive to price promotions on less healthy rather than healthier products [16], and that consumers may be more responsive to price discounts on less healthy foods and price increases on healthier foods [17]. Experimental studies looking at changing the proximity of foods have altered both healthier and less healthy foods, and have not suggested any differences by food healthiness [18, 19] – however, these have focused on altering just one example of a healthier and less healthy food. No experimental studies to our knowledge have set out to isolate responses to altering a range of healthier vs. less healthy foods, which is likely to better reflect many food environments.

This distinction between healthier and less healthy food cues may also have implications for socioeconomic inequalities. Living in differentially ‘obesogenic’ environments may drive some of the socioeconomic differences in diet-related behaviours, e.g. those who are more deprived may have less exposure to healthier environmental cues, such as the presence of healthier food outlets, and greater exposure to less healthy environmental cues, such as unhealthy food outlets [20–22]. How people respond to the same environmental cues may additionally contribute to inequalities: response inhibition (a core element of executive function that includes being able to resist impulsive behaviour [23]) is associated with socioeconomic status (SES) [24, 25], and predicts obesity and food-related behaviour [26–29]. The healthiness of the food involved may play a role, however, with response inhibition having a more limited (if any) impact on consumption of healthier foods [30–32]. As such, the choice of targeting healthier or less healthy food cues may have implications for the effectiveness of an intervention across socioeconomic groups, and any differential responsiveness is essential to establish in order to select interventions for implementation that will not inadvertently increase inequalities.

Given the association between socioeconomic status and response inhibition [24, 25], it is interesting to investigate people’s responses to food cues when their response inhibition has been lowered. One means of targeting response inhibition is increasing cognitive load, which can be used to temporarily deplete an individual’s cognitive resources (including response inhibition) [33–35]. The effect of increasing cognitive load is also worthy of exploration in the context of making changes to environmental cues, such as product availability, given that it has been hypothesised the changes to the physical micro-environment may impact on behaviour without relying on individuals’ cognitive resources [3, 9]. Moreover the effects of increasing cognitive load when exploring cues targeting healthier vs. less healthy foods have not been explored to our knowledge, and may vary given the different associations between response inhibition and healthier/ less healthy food choices.

It is worth noting that any effects of response inhibition may also be moderated by food appeal – with those with strong appeal towards less healthy foods and lower response inhibition being more likely to make less healthy choices and to gain the most weight [36–39]. This may also have further implications for socioeconomic inequalities, as some healthier foods have higher appeal for less deprived individuals [40]. As such, food appeal may act alongside response inhibition to mediate some of the socioeconomic patterning seen in diet-related behaviour, and may contribute to any differences in responses to healthier and less healthy food cues.

To address some of the gaps in the extant literature, the current study aims to examine: (a) the impact of increasing the range of (i) healthier (i.e. lower energy) snack foods vs. (ii) less healthy (i.e. higher energy) snack foods on food selection in an online task; and the potential moderation of responses to these cues by (b) cognitive load and (c) by socioeconomic status. In addition, response inhibition and food appeal will be investigated as potential mediators of any influence of socioeconomic status on food choice. Snack foods (operationalized as single-serve pre-packaged foods, including confectionery, potato chips and cereal bars) were chosen as an initial category to investigate this hypothesis, given they are more likely to be selected and consumed within a short interval, potentially making them more susceptible to fluctuations in response inhibition than meals. The specific hypotheses tested are set out below.

### Primary hypothesis

1. Increasing the number of less healthy food items has a larger effect on the healthiness of food choices than increasing the number of healthier food items

### Secondary hypotheses

2. Cognitive load: Participants under high (vs low) cognitive load:
  - will show no differences in their likelihood of selecting healthier foods after seeing a greater number of healthier food options
  - will be more likely to select less healthy foods after seeing a greater number of less healthy food options
3. Socioeconomic status: Participants with higher (vs lower) socioeconomic status:
  - will be more likely to select healthier foods after seeing a greater number of healthier food options
  - will be less likely to choose less healthy foods after seeing a greater number of less healthy food options
4. Response inhibition and food appeal both partially mediate the impact of socioeconomic status on food choice

## Methods

### Design

Participants were randomly allocated to one of six groups in a between-subjects design (three availability conditions x two cognitive load conditions). Randomisation was conducted online using the Qualtrics randomiser element, and was performed separately for each of three socioeconomic groups (defined by occupational group), to achieve similar numbers of participants of each socioeconomic status in each study group. As such, neither the recruiter nor researcher were aware of participants’ group assignment prior to participation.

#### Availability conditions

Participants were asked to select an item from an array of snack foods that they would most like to eat right now. The composition of this array differed between participants depending on their assignment to one of three conditions: (1) two healthier and two less healthy food items (reference); (2) two healthier and six less healthy food items (increased less healthy); (3) six healthier and two less healthy food items (increased healthier). As such, comparing condition 2 to condition 1 involved changing the number of less healthy items while keeping the number of healthier items constant (and vice versa comparing condition 3 to condition 1). The intervention also involved changing the proportion of healthier to less healthy and the overall number of options, but this was mirrored across the two conditions where options were increased (conditions 2 and 3).

#### Cognitive load conditions

Participants were asked to memorise a 7-digit number as part of the study. They were randomised to either a complex string (e.g. 8529713; high load) or simple string (e.g. 7777777; low load).

The study was pre-registered on the Open Science Framework (https://osf.io/nxt4s/), and ethical approval was obtained from the Cambridge Psychology Research Ethics Committee (Pre.2017.016).

### Sample

The sample of 1509 UK adults was recruited from an online market research company panel (Research Now). No specific inclusion criteria were used, but the sample was selected to be representative of the UK in terms of age and gender, with quotas set for socioeconomic status (evenly divided between occupational status groups A&B: Higher and intermediate managerial, administrative and professional occupations; C1&C2: Supervisory, clerical and junior managerial, administrative and professional occupations; D&E: Semi-skilled and unskilled manual occupations). Participants are paid in vouchers for their time spent completing surveys for the market research company, with participation in this study being paid at the usual rate.

The planned sample size was determined using G*Power (version 3.1.9.2), for a logistic regression, with power of 0.8 and alpha =0.025, to detect a small effect size (odds ratio 1.5) using a binomial predictor variable, with balanced groups. The effect size was based on the impact of availability on food choice in pilot work and the r-squared accounted for by control variables was taken to be medium-sized (0.25). This gives a sample estimate of 1257, for a 2-group comparison (i.e. 629 per group, which was rounded to 630 to give a slight over-recruitment). For the 3 availability conditions × 2 cognitive load conditions, this gave a total sample size of 3780.

However, due to issues with recruitment from the online panel, the total sample size could not be achieved. When this became apparent, recruitment was paused and a post-hoc internal pilot was conducted to determine whether additional data should be sought from an alternative source. The data obtained thus far was given to a statistician (who was not responsible for the study analyses), who conducted an updated sample size estimate, based on the actual effect size. This revised estimate suggested that a total sample size of 579 would allow a test the impact of availability on food choice with a power of 0.8. Given this sample had already been achieved, recruitment was halted.

#### Exclusions

Anyone completing the survey in less than 30% of the median time was excluded (no participants met this exclusion criteria). In addition, participants had to correctly answer a quality control question as part of the study to ensure that they are paying attention to the questions (“How many times have you visited the planet Mars?”). Anyone answering incorrectly (i.e. any answer other than “Never”) was screened out and was not counted towards the study quotas (*n* = 321).

### Measures

#### Outcome: Food choice (healthier or less healthy)

Participants’ choice of a healthier or less healthy snack food was the main study outcome. Participants were asked which of an array of items they would most like to eat right now, with the array differing depending on their assigned availability condition.

##### Healthier vs. less healthy foods

The study focused on pre-packaged snack food, with the relative healthiness of snack foods defined by kcal per pack. While this does not encompass the full picture with regard to healthier diets, reducing the energy consumed from discretionary foods like snacks – which tend to have limited nutritional value [41] – is a relevant public health target, given adults on average consume 200 kcal per day over their recommended energy intake in the UK [42].

Healthier snack foods: 100 kcal or less per pack. The 100 kcal limit was chosen based on Change4Life’s recommendation of a 400 kcal per day allowance for snacks and drinks [43], dividing this into a 100 kcal allowance for two snacks and two drinks.

Less healthy snack foods: 200 kcal or more per pack. This would then mean that consuming two of these snacks daily would exceed Change4Life’s recommended allowance, without considering drinks.

##### Piloting of healthy and less healthy food choices

A pilot survey was conducted to choose the snack foods to use in the main study. One hundred UK adults were recruited by the same market research company, with equal quotas by the same three occupational groups. Participants were presented with pictures of food items (front-of-pack only), and asked to rate these on familiarity, appeal, serving size and healthiness.

This pilot work identified a selection of six healthier and six less healthy snack foods whereby:

- Healthier items all had higher mean perceived healthiness scores than any of the less healthy items;
- Healthier and less healthy foods were matched in terms of perceived familiarity;
- All packages were perceived as single-serve.

The six healthier options were Alpen Light Chocolate and Fudge bar (19 g), Special K Red Berry Cereal bar (21.5 g), Nakd Banana Bread bar (30 g), Walkers Pops Original (19 g), Sunbites Lightly Salted Popcorn (20 g) and Kettle Bites Maple Barbeque Waves (22 g). The six less healthy options were: Reese’s Snack Mix (56 g), Dairy Milk Big Taste Toffee Whole Nut bar (43 g), Niknaks Nice ‘N’ Spicy (50 g), Kettle Chips Crispy Bacon and Maple Syrup (40 g), Lindt Lindor Milk Chocolate Orange bar (38 g), Walkers Max Paprika (50 g).

Food items were not matched on appeal, given that food appeal may vary between healthier and less healthy foods, and may mediate some of the pathway between socioeconomic status and food choice.

#### Socioeconomic status

This was assessed via four indicators: (1) occupational group; (2) highest educational qualification, (3) total annual household income, and (4) Index of Multiple Deprivation scores.

Participants’ occupational group was provided by the market research company. In addition, participants were asked to indicate their highest educational qualification and total annual household income (see Table 1 for the categorisations used). Index of Multiple Deprivation scores were derived from participants’ postcodes (using adjusted indices to account for participants being from different parts of the UK [44]).

**Table 1:** Study group allocation and characteristics of participants (% (n))

Study group	Equal healthier/ less healthy item availability	High cognitive load	17.0% (257)
		Low cognitive load	15.4% (233)
	Increased healthier item availability	High cognitive load	17.4% (263)
		Low cognitive load	16.1% (243)
	Increased less healthy item availability	High cognitive load	16.8% (254)
		Low cognitive load	17.2% (259)
Socioeconomic status	Occupational group^a^	A&B	34.0% (513)
		C1&C2	33.3% (503)
		D&E	32.7% (493)
	Highest educational qualification	1–4 GCSEs or equivalent	10.7% (162)
		5+ GCSEs or equivalent	15.6% (236)
		2+ A Levels or equivalent	9.3% (141)
		Degree or equivalent, or higher	46.7% (704)
		Prefer not to answer/ Still studying/ Foreign qualifications	17.6% (266)
	Annual household income (GBP)	Up to £17,499	25.8% (389)
		£17,500–£29,999	24.5% (370)
		£30,000–£49,999	27.0% (408)
		£50,000+	19.2% (290)
		Prefer not to answer/ Don’t know	3.4% (52)
	Index of Multiple Deprivation quintiles^b^	Quintile 1: Least deprived	18.6% (281)
		Quintile 2	18.6% (281)
		Quintile 3	19.2% (289)
		Quintile 4	18.7% (282)
		Quintile 5: Most deprived	18.6% (281)
		Prefer not to answer/ Postcode not recognised	6.3% (95)
Cognitive load	Percentage recalling number correctly	Low load	97.8% (719/735)
		High load	74.4% (576/774)
Food appeal	Mean enjoyment ratings^c^	Healthier *Mean (s.d.)*	0.56 (1.06)
		Less healthy *Mean (s.d.)*	0.99 (1.09)
Response inhibition	SUPPS-P	*Mean (s.d.)*	40.8 (9.5)
Hunger	Mean hunger rating	*Mean (s.d.)*	0.33 (1.37)

^a^A&B: Higher and intermediate managerial, administrative and professional occupations; C1&C2: Supervisory, clerical and junior managerial, administrative and professional occupations; D&E: Semi-skilled and unskilled manual occupations

^b^Quintiles defined within sample, with IMD scores of: Least deprived: 0.98 to 8.25; 2: 8.26 to 13.73, 3: 13.77 to 20.68; 4: 20.7 to 32.75, Most deprived: 32.87 to 77.29

^c^A rating of 0 was labelled ‘Neither enjoyable nor unenjoyable’, and 1 ‘Quite enjoyable’

#### Food appeal

Participants were presented with pictures of snack foods, including those used in the food choice task, and rated “How enjoyable is eating this food?” using a seven-point scale from *Unenjoyable – Enjoyable* (e.g. [45]). The order in which pictures were presented was randomised.

#### Response inhibition

The Short-form UPPS-P Impulsive Behavior Scale (SUPPS-P [46]) was used as a trait measure of impulsivity. The order in which items were presented was randomised.

### Procedure

Members of the market research panel were sent a link to the study website, where the study was described as investigating the appeal of snack food. After consenting to the study, they rated pictures of snack foods for enjoyability (food appeal) and completed the SUPPS-P (response inhibition). The quality control question was embedded within the picture rating section; participants answering this incorrectly were screened out of the survey. Following this, participants were randomised to one of six groups (two cognitive load conditions x three availability conditions). All participants were asked to memorise a 7-digit number (either a complex string for high cognitive load or a simple string for low cognitive load). Participants needed to press two keys (‘Q’ and ‘P’) simultaneously to reveal the number (to discourage cheating on this task, given it was conducted online), which was displayed for 10 s (using Inquisit Web). Participants were then shown an array of food items on screen, and asked to select the item that they would most like to eat right now. The image for each item displayed the front-of-pack only. Each participant saw a single array, from which they were able to select one item. The number of healthier and less healthy foods in the array differed depending on their assigned availability condition. The food items offered to each participant were selected at random from the pool of healthier and less healthy items, and their positions in the array were also randomly determined (operationalised in Inquisit by setting the selection mode to random). Following the food choice, participants were asked to recall the 7-digit number that they had memorised. Finally participants completed a set of demographic questions, including socioeconomic status measures, and hunger ratings (using a 7-pt rating scale from Very hungry – Very full).

#### Changes from study pre-registration

Two changes were made after pre-registration of this study (https://osf.io/nxt4s/):

- Firstly, the sample size was reduced, as outlined above, due to problems with recruitment
- Secondly, due to concerns about the length of the survey, the planned implicit measures of food appeal and response inhibition were moved to a secondary study session, and as such are not reported here. We used the explicit measures of food appeal and response inhibition described here to explore our hypotheses with regard to these variables.

### Analysis

#### Primary hypothesis

Hypothesis 1 (*Increasing the number of less healthy food items has a larger effect than increasing the number of healthier food items*):

This was analysed via logistic regression (using Stata SE version 12.1) predicting choice of a healthier food option, with dummy variables indicating the availability and cognitive load conditions as the key predictors. For availability, the two healthier & two less healthy choices condition was the reference group, with two dummy variables for the other availability conditions indicating (1) increase in healthier options and (2) increase in less healthy options. For cognitive load, a dummy variable indicating high load was used. Control variables included socioeconomic status, gender, age and hunger.

#### Secondary hypotheses

Hypotheses 2a & 2b: Cognitive load (*Participants under high cognitive load will not be significantly more or significantly less likely to choose healthier foods after seeing a greater number of healthier food options than those under low cognitive load; Participants under high cognitive load are more likely to choose less healthy foods after seeing a greater number of less healthy food options than those under low cognitive load*):

Interactions between availability condition and cognitive load were added to the model used for hypothesis 1. That is, dummy variables indicating (1) high_cognitive_load* increase_in_healthier_options; (2) high_cognitive_load* increase_in_less_healthy_options.

Hypotheses 3a & 3b: Socioeconomic status (*Participants with higher socioeconomic status are more likely to choose healthier foods after seeing a greater number of healthier food options than those with lower socioeconomic status; Participants with higher socioeconomic status are less likely to choose less healthy foods after seeing a greater number of less healthy food options than those with lower socioeconomic status*):

Interactions between availability condition and socioeconomic status (separately for each of the four indicators) were added to the model used for hypothesis 1. Socioeconomic patterning was examined for each different measure, given that these indices are thought to be conceptually distinct, and have different pathways of influence. Each socioeconomic indicator was modelled as a set of dummy variables, using the categorisations shown in Table 1.

For example, for the occupational group socioeconomic status indicator, with occupational group A&B as the reference, these interactions were dummy variables indicating (1) occupational_group_C1&C2* increase_in_healthier_options; (2) occupational_group_D&E* increase_in_healthier_options; (3) occupational_group_C1&C2* increase_in_less_healthy_options; (4) occupational_group_D&E* increase_in_less_healthy_options.

Hypothesis 4 (*Response inhibition and food appeal both partially mediate the impact of socioeconomic status on food choice*):

If the analyses in (1) and (3) suggested a relationship between socioeconomic status and food choice, separate mediation analyses were planned to investigate the extent to which (a) response inhibition variables and (b) food appeal variables mediate any relationship between socioeconomic status (each indicator separately) and food choice.

For our primary hypothesis (hypothesis 1), we used *p* < 0.05 (two-tailed) to infer if there was a statistically significant effect. For the remaining analyses (secondary hypotheses regarding interactions and mediators), we used a *p*-value < 0.0027 (two-tailed), using a Bonferroni adjustment to account for the different hypotheses tested and analyses by different SES indicators (*p* = 0.05/18).

## Results

Table 1 shows the study group allocation and characteristics of the 1509 study participants. Their mean age was 49.6 (s.d. 15.4; range 18–92), and 46.6% identified as female (the remainder identifying as male).

**Fig. 1:**
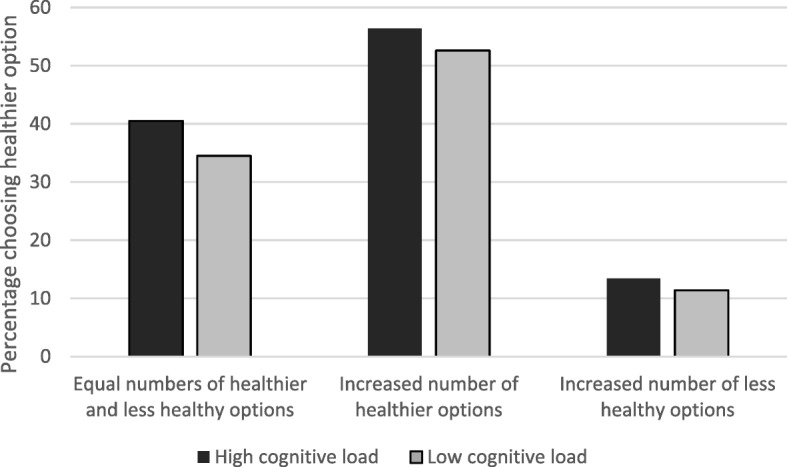
Percentage choosing healthier option by cognitive load condition

Figure 1 shows the percentage of participants choosing a healthier option, broken down by cognitive load condition (see Additional file 1: Table S1 for numbers in each group). The pattern of results here suggests a strong effect of availability (55% choosing a healthier item with increased healthier options vs. 38% with equal options vs. 12% with increased less healthy options), with limited impact apparent by cognitive load condition.

### Hypothesis 1

**Fig. 2:**
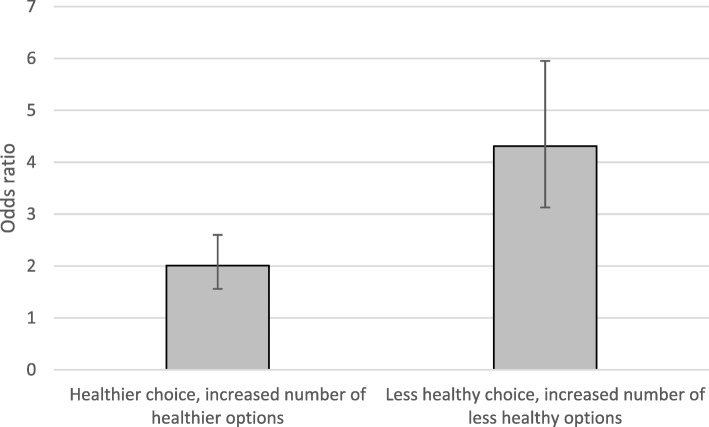
Effects of increasing healthier vs. less healthy options on food choice: Odds ratios (and 95% CIs) of making a healthier (less healthy) choice with increased numbers of healthier (less healthy) options, relative to having equal numbers of healthier and less healthy options

Figure 2 presents the results of logistic regressions used to test the impact of availability for healthier vs. less healthy food options (Hypothesis 1; see Additional file 1: Table S2 for full results). The odds of choosing a healthier option are twice as high (OR: 2.0, 95%CI: 1.6, 2.6) when offered six healthier options and two less healthy, than when offered two healthier and two less healthy options. The odds of choosing a less healthy option (i.e. reversing the outcome measure to obtain comparable odds ratios) are four times higher (OR: 4.3, 95%CI: 3.1, 6.0) when offered two healthier options and six less healthy, than when offered two healthier and two less healthy options. Comparing these two odds ratios (using Stata’s ‘contrast’ command), the odds of making a less healthy choice after seeing an increased number of less healthy options are 2.16 times higher (95%CI: 1.8, 2.5) than the odds of a healthier choice after seeing an increased number of healthier options.

### Hypothesis 2

No significant main effects of cognitive load, or interactions between cognitive load and availability condition, were shown in analyses of food choice (see Additional file 1: Table S3).

### Hypothesis 3

**Fig. 3:**
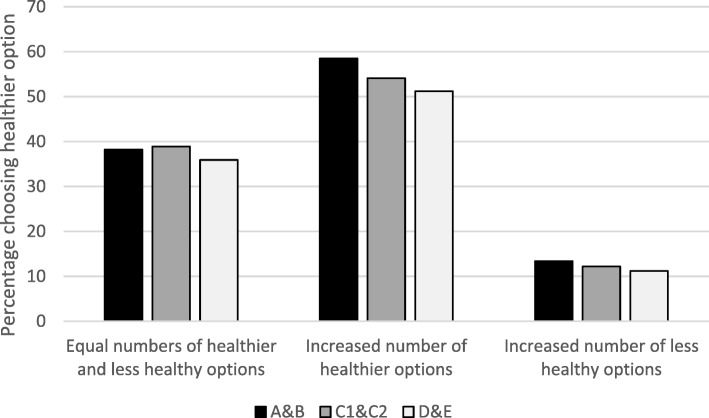
Percentage choosing healthier options by occupational group^1^. ^1^ A&B: Higher and intermediate managerial, administrative and professional occupations; C1&C2: Supervisory, clerical and junior managerial, administrative and professional occupations; D&E: Semi-skilled and unskilled manual occupations

Figure 3 shows the percentage choosing healthier options by occupational group. Regression analyses found no significant differences in choosing a healthier option across socioeconomic status using any of the four measures examined. Similarly, no interactions between any measure of socioeconomic status and availability condition were shown in analyses (see Additional file 1: Tables S4a-d).

### Hypothesis 4

Given the expected socioeconomic patterning in food choice was not found, the planned mediation analysis for hypothesis 4 was not applicable. Exploratory regression analyses (using *p*-value < 0.002, to adjust for the additional comparisons) instead examined the two halves of the pathway in the proposed mediation, i.e. (1) whether socioeconomic status predicted (i) food appeal and (ii) response inhibition; and (2) whether (i) food appeal and (ii) response inhibition predicted food choice.

1. Analyses showed no significant differences by any measure of socioeconomic status for either food appeal or response inhibition (see Additional file 1: Table S5).
2. Both enjoyment ratings (food appeal) but not SUPPS-P scores (response inhibition) predicted food choice (see Additional file 1: Table S6), with higher odds of participants choosing a healthier option if they had less liking for less healthy snacks (OR: 0.41, 95%CI: 0.35, 0.49) or greater liking for healthier snacks (OR: 2.17, 95%CI: 1.84, 2.57)).

## Discussion

The results of this study suggest that altering the availability of less healthy food may have more impact on the healthiness of food choices than altering the availability of healthier food, supporting Hypothesis 1. Indeed, the odds of making a less healthy choice after seeing an increased number of less healthy options were twice as high as the odds of a healthier choice after seeing an increased number of healthier options. This is one of the first experimental studies to explore the relative effectiveness of healthier vs. less healthy food cues at influencing behaviour.

These results tie in with previous observational research looking at both food availability and price [15–17], which suggested people may be more responsive to cues encouraging less healthy food choices. This may in part reflect differential appeal of healthier and less healthy items (less healthy items were rated as more enjoyable to eat in the current study), with people perhaps being more responsive to cues for foods they find more appealing. That said, in the exploratory analyses the effects of availability did not change when enjoyment ratings for healthier and less healthy snack foods were included in models. While previous experimental studies examining the effect of proximity of healthier and less healthy foods have not suggested differential responsiveness [18, 19], the current study set-up involves both a wider range of food items and an explicit choice (rather than being able to select both), which may allow an effect of food healthiness to be more readily observed.

The study also explored additional hypotheses relating to the potential for cognitive load or socioeconomic status to modify the impact of these different food cues. However, the results suggested no significant main effects on food choice, or interactions with availability condition for either cognitive load or socioeconomic status. As such, the other hypothesised relationships were not supported by these analyses.

In terms of cognitive load, the two main explanations for the effects found here are that the manipulation we used was not effective, or that it was effective but the choices made were not affected by cognitive load. It cannot be discounted that the manipulation may not have impacted on cognitive load as strongly as expected, as it was not possible to include a manipulation check due to concerns about survey length. Given that Shiv and Fedorikhin found that the effect of cognitive load in their study was only apparent when actual foods were presented [33], repeating this element of the study with choices between physically-present foods would be valuable. Nevertheless, the lack of effect may indicate that cognitive load did not influence people’s choice of food, as has previously been demonstrated in studies of food proximity [47]. This could suggest that altering the availability of healthier and less healthy food impacts behaviour without requiring cognitive resource, such as response inhibition, as has been hypothesised for interventions targeting physical micro-environments [3, 9]. If so, then this could mean that this intervention is likely to be effective regardless of people’s current cognitive resources, which would be promising in terms of the potential for such interventions to change behaviour across socioeconomic groups.

While socioeconomic patterning in diet is well documented [48–50], this does vary by food type [51]. The results here suggest that the snack foods used in the current study may represent a set of food for which appeal and choice does not differ across socioeconomic groups. As such, if there is any differential response to certain types of food cue by socioeconomic status, driven in part by differential response inhibition or food appeal, this would not be picked up in the current study. On the other hand, if the lack of social patterning seen here in responses to the intervention does prove consistent across food types, this would suggest that interventions targeting food availability would be unlikely to widen health inequalities.

In terms of the potential for response inhibition and food appeal to mediate differences in food choice by socioeconomic group, exploratory analyses suggested that food appeal (but not response inhibition) predicted food choice in the current investigation. While these results support food appeal as a potential driver of diet-related behaviour, the lack of effect of response inhibition is in contrast to those seen in previous studies [26–29]. However, given that the SUPPS-P is a trait-level measure, this may reflect that while measures such as the SUPPS-P might predict aggregated food choices over time, they may not be discriminatory for a one-off task.

### Strengths and limitations

This study offers a novel test of the relative impact of increasing healthier vs. less healthy food cues, matching healthier and less healthy food items on familiarity and controlling for the number of each. The study was conducted using a large sample, broadly representative of the UK in terms of age and gender, and with quotas ensuring equal representation by occupational status. It should be noted, however, that this did not equate to the sample being representative across all socioeconomic indicators, with the sample being more highly educated than the UK as a whole. Nonetheless, this study provides some of the most robust evidence to date that there may be a stronger impact of reducing less healthy food cues than increasing – by an equivalent number – healthier food cues.

However, several limitations to the study should be noted. Firstly, as this was an online study, the food choice task did not include selection with physically present foods or consumption. This can be addressed in subsequent studies using a food choice task in which participants receive the food item in question. Secondly, it was not possible to include a manipulation check for the cognitive load manipulation due to concerns over study length. While other studies have used a similar task to manipulate cognitive load [33–35], uncertainty remains in the current context. Thirdly, while focusing on only a small number of food items was necessary for this online food choice task, this limited the potential to examine socioeconomic patterning, seen when looking across diets. Indeed, investigating a wider range of foods, including items that have a healthier nutritional profile overall – rather than focusing only on lower energy items – would be a valuable extension to this study. Finally, including implicit (state rather than trait) measures of food appeal and response inhibition concurrent with the food choice task would strengthen testing of these potential pathways, in particular, for response inhibition.

### Implications for research and policy

These results require replication, in particular, in real world settings used by those who are more and less socially deprived, and altering availability in additional ways such as changing the number but not the range of options. If replicated, the greater impact of less healthy food cues compared to healthier food cues would prioritise removing less healthy cues over adding healthier cues in policies for healthier eating. Further research could also explore the potential for differential effects by socioeconomic status through examining a broader range of foods or food types for which consumption is known to be socially patterned. Establishing which cues are most influential on behaviour, and in particular which have the greatest impact on more socially deprived groups, could help in designing more effective public health interventions to reduce both the substantial burden of non-communicable diseases and their contribution to health inequalities.

## Conclusion

This study provides a novel test of the relative impact of healthier vs. less healthy food cues on food choice, suggesting that less healthy food cues may be more influential. Further work is required to try to replicate these findings in experiments requiring participants to make choices between physically-present food items, and when using different ways of altering availability, as well as to explore the potential for differential effects by socioeconomic status using other food options. If replicated, the greater impact of less healthy food cues than healthier food cues should prioritise a healthier eating policy focus on reducing less healthy food cues rather than increasing healthier cues.

## Additional file

Additional file 1: **Table S1.** Percentage (n) choosing healthier option. **Table S2.** Results of logistic regression predicting healthier food choice (less healthy food choice for alternative outcome row) from availability conditions, controlling for age, gender, hunger, social class. **Table S3.** Results of logistic regression predicting healthier food choice from availability conditions, with interactions by cognitive load. **Tables S4a-d.** Results of logistic regressions predicting healthier food choice from availability conditions, with interactions by socioeconomic status. **Table S5.** Regression coefficients predicting (i) enjoyment of healthier snack options, (ii) enjoyment of less healthy snack options and (iii) SUPPS-P total scores from socioeconomic status, controlling for availability condition, cognitive load condition, gender, age and hunger. **Table S6.** Results of logistic regression predicting healthier food choice from availability conditions, with enjoyment ratings and impulsivity (SUPPS-P) as predictors. (DOCX 42 kb)

## Abbreviations

BMI: Body mass index
NCDs: Non-communicable diseases
OR: Odds ratio
SES: Socioeconomic status
SUPPS-P: Short-form Urgency, Premeditation (lack of), Perseverance (lack of), Sensation Seeking, Positive Urgency, Impulsive Behavior Scale
