# Supplementary material for: Availability of healthier vs. less healthy food and food choice: an online experiment
Source: BMC Public Health. 2018 Nov 29;18:1296. doi: 10.1186/s12889-018-6112-3 (PMC6264049; doi:10.1186/s12889-018-6112-3)
Supplement: Supplementary file 1 — Table S1. Percentage (n) choosing healthier option. Table S2. Results of logistic regression predicting healthier food choice (less healthy food choice for alternative outcome row) from availability conditions, controlling for age, gender, hunger, social class. Table S3. Results of logistic regression predicting healthier food choice from availability conditions, with interactions by cognitive load. Tables S4a-d. Results of logistic regressions predicting healthier food choice from availability conditions, with interactions by socioeconomic status. Table S5. Regression coefficients predicting (i) enjoyment of healthier snack options, (ii) enjoyment of less healthy snack options and (iii) SUPPS-P total scores from socioeconomic status, controlling for availability condition, cognitive load condition, gender, age and hunger. Table S6. Results of logistic regression predicting healthier food choice from availability conditions, with enjoyment ratings and impulsivity (SUPPS-P) as predictors. (DOCX 42 kb) [file 12889_2018_6112_MOESM1_ESM.docx]

**Additional file 1**

**Table S1.** Percentage (n) choosing healthier option

|  | *Offered an increased number of healthier options* | *Offered an increased number of less healthy options* | *Offered equal numbers of healthier and less healthy options* | *Total* |
| --- | --- | --- | --- | --- |
| High cognitive load | 56.4  (147) | 13.4  (34) | 40.5  (105) | 37.0  (238) |
| Low cognitive load | 52.6  (129) | 11.4  (29) | 34.5  (80) | 32.4  (286) |
|  | | | | |
| A&B | 58.5  (96) | 13.4  (24) | 38.2  (65) | 36.1  (185) |
| C1&C2 | 54.1  (93) | 12.2  (20) | 38.9  (65) | 35.4  (178) |
| D&E | 51.2  (87) | 11.2  (19) | 35.9  (55) | 32.7  (161) |
|  | | | | |
| Total | 54.5  (276) | 12.3  (63) | 37.8  (185) | 34.7  (524) |

**Table S2.** Results of logistic regression predicting healthier food choice (less healthy food choice for alternative outcome row) from availability conditions, controlling for age, gender, hunger, social class

|  | | *Odds Ratio* | *Std. Err.* | *P* | *95% Conf. Interval* | |
| --- | --- | --- | --- | --- | --- | --- |
| Availability  (Ref: Equal) | Healthier | 2.01 | 0.26 | 0.00 | 1.56 | 2.60 |
|  | Less healthy | 0.23 | 0.04 | 0.00 | 0.17 | 0.32 |
| High cognitive load | | 1.21 | 0.14 | 0.10 | 0.96 | 1.53 |
| Age | | 1.01 | 0.00 | 0.14 | 1.00 | 1.01 |
| Female | | 1.29 | 0.16 | 0.04 | 1.02 | 1.64 |
| Hunger | | 0.96 | 0.04 | 0.33 | 0.88 | 1.04 |
| Social class  (Ref: A&B) | C1&C2 | 0.92 | 0.13 | 0.56 | 0.70 | 1.22 |
|  | D&E | 0.80 | 0.12 | 0.12 | 0.60 | 1.06 |
| Constant | | 0.40 | 0.11 | 0.00 | 0.24 | 0.68 |
| *Alternative outcome: Choosing less healthy option* | | | | | | |
| Availability  (Ref: Equal) | Healthier | 0.50 | 0.06 | 0.00 | 0.39 | 0.64 |
|  | Less healthy | 4.31 | 0.71 | 0.00 | 3.13 | 5.95 |

Pseudo R-squared: 0.12, Hosmer-Lemeshow chi-square 4.72, p=0.79

**Table S3**. Results of logistic regression predicting healthier food choice from availability conditions, with interactions by cognitive load.

|  | | *Odds Ratio* | *Std. Err.* | *P* | *95% Conf. Interval* | |
| --- | --- | --- | --- | --- | --- | --- |
| Availability  (Ref: Equal) | Healthier | 2.19 | 0.42 | 0.00 | 1.51 | 3.18 |
|  | Less healthy | 0.24 | 0.06 | 0.00 | 0.15 | 0.39 |
| High cognitive load | | 1.32 | 0.25 | 0.14 | 0.91 | 1.91 |
| High cognitive load*Healthier availability | | 0.85 | 0.22 | 0.53 | 0.51 | 1.41 |
| High cognitive load*Less healthy availability | | 0.93 | 0.31 | 0.83 | 0.49 | 1.78 |
| Social class  (Ref: A&B) | C1&C2 | 0.92 | 0.13 | 0.56 | 0.70 | 1.22 |
|  | D&E | 0.80 | 0.12 | 0.12 | 0.60 | 1.06 |
| Age | | 1.01 | 0.00 | 0.15 | 1.00 | 1.01 |
| Female | | 1.29 | 0.16 | 0.04 | 1.02 | 1.64 |
| Hunger | | 0.96 | 0.04 | 0.34 | 0.88 | 1.04 |
| Constant | | 0.39 | 0.11 | 0.00 | 0.22 | 0.67 |

Pseudo R-squared: 0.12, Hosmer-Lemeshow chi-square 3.14, p=0.95

**Tables S4a-d**. Results of logistic regressions predicting healthier food choice from availability conditions, with interactions by socioeconomic status.

1. Socioeconomic status defined by occupational group:

|  | | *Odds Ratio* | *Std. Err.* | *P* | *95% Conf. Interval* | |
| --- | --- | --- | --- | --- | --- | --- |
| Availability  (Ref: Equal) | Healthier | 2.32 | 0.52 | 0.00 | 1.49 | 3.61 |
|  | Less healthy | 0.25 | 0.07 | 0.00 | 0.15 | 0.43 |
| Social class  (Ref: A&B) | C1&C2 | 1.03 | 0.23 | 0.89 | 0.66 | 1.60 |
|  | D&E | 0.90 | 0.21 | 0.66 | 0.57 | 1.42 |
| Healthier availability * C1&C2 | | 0.81 | 0.26 | 0.51 | 0.44 | 1.51 |
| Healthier availability * D&E | | 0.80 | 0.26 | 0.48 | 0.43 | 1.50 |
| Less healthy availability * C1&C2 | | 0.86 | 0.34 | 0.69 | 0.39 | 1.86 |
| Less healthy availability * D&E | | 0.87 | 0.35 | 0.73 | 0.40 | 1.92 |
| High cognitive load | | 1.21 | 0.14 | 0.10 | 0.97 | 1.53 |
| Age | | 1.01 | 0.00 | 0.15 | 1.00 | 1.01 |
| Female | | 1.29 | 0.16 | 0.04 | 1.02 | 1.64 |
| Hunger | | 0.96 | 0.04 | 0.32 | 0.88 | 1.04 |
| Constant | | 0.38 | 0.11 | 0.00 | 0.21 | 0.66 |

Pseudo R-squared: 0.12, Hosmer-Lemeshow chi-square 5.05, p=0.75

1. Socioeconomic status defined by highest educational qualification:

|  | | *Odds Ratio* | *Std. Err.* | *P* | *95% Conf. Interval* | |
| --- | --- | --- | --- | --- | --- | --- |
| Availability  (Ref: Equal) | Healthier | 2.72 | 1.16 | 0.02 | 1.18 | 6.27 |
|  | Less healthy | 0.36 | 0.19 | 0.05 | 0.13 | 0.99 |
| Highest educational qualification (Ref: 1-4 GCSEs) | 5+ GCSEs | 1.71 | 0.69 | 0.19 | 0.77 | 3.78 |
|  | 2+ A Levels | 1.44 | 0.64 | 0.41 | 0.61 | 3.45 |
|  | Degree or higher | 1.81 | 0.64 | 0.09 | 0.91 | 3.61 |
| Healthier availability * 5+ GCSEs | | 0.61 | 0.33 | 0.36 | 0.21 | 1.75 |
| Healthier availability * 2+ A Levels | | 0.99 | 0.60 | 0.99 | 0.31 | 3.23 |
| Healthier availability * Degree or higher | | 0.64 | 0.30 | 0.34 | 0.26 | 1.59 |
| Less healthy availability * 5+ GCSEs | | 0.43 | 0.30 | 0.23 | 0.11 | 1.69 |
| Less healthy availability * 2+ A Levels | | 0.24 | 0.23 | 0.13 | 0.04 | 1.53 |
| Less healthy availability * Degree or higher | | 0.77 | 0.43 | 0.64 | 0.25 | 2.32 |
| High cognitive load | | 1.13 | 0.15 | 0.36 | 0.87 | 1.45 |
| Age | | 1.00 | 0.00 | 0.38 | 1.00 | 1.01 |
| Female | | 1.30 | 0.18 | 0.05 | 1.00 | 1.69 |
| Hunger | | 1.00 | 0.05 | 0.97 | 0.91 | 1.10 |
| Constant | | 0.25 | 0.11 | 0.00 | 0.11 | 0.57 |

Pseudo R-squared: 0.12, Hosmer-Lemeshow chi-square 1.77, p=0.99

1. Socioeconomic status defined by income group:

|  | | *Odds Ratio* | *Std. Err.* | *P* | *95% Conf. Interval* | |
| --- | --- | --- | --- | --- | --- | --- |
| Availability  (Ref: Equal) | Healthier | 2.00 | 0.51 | 0.01 | 1.22 | 3.28 |
|  | Less healthy | 0.23 | 0.08 | 0.00 | 0.12 | 0.45 |
| Income group (Ref: Up to £17,499) | £17,500-£29,999 | 1.08 | 0.29 | 0.77 | 0.64 | 1.82 |
|  | £30,000-£49,999 | 1.36 | 0.35 | 0.24 | 0.82 | 2.26 |
|  | £50,000+ | 1.16 | 0.32 | 0.59 | 0.68 | 1.98 |
| Healthier availability * £17,500-£29,999 | | 0.86 | 0.32 | 0.69 | 0.42 | 1.78 |
| Healthier availability * £30,000-£49,999 | | 0.90 | 0.32 | 0.77 | 0.45 | 1.80 |
| Healthier availability * £50,000+ | | 1.18 | 0.46 | 0.67 | 0.55 | 2.52 |
| Less healthy availability * £17,500-£29,999 | | 1.30 | 0.60 | 0.56 | 0.53 | 3.19 |
| Less healthy availability * £30,000-£49,999 | | 0.75 | 0.35 | 0.53 | 0.30 | 1.87 |
| Less healthy availability * £50,000+ | | 0.78 | 0.41 | 0.65 | 0.28 | 2.21 |
| High cognitive load | | 1.16 | 0.14 | 0.20 | 0.92 | 1.47 |
| Age | | 1.01 | 0.00 | 0.14 | 1.00 | 1.01 |
| Female | | 1.29 | 0.16 | 0.04 | 1.01 | 1.65 |
| Hunger | | 0.93 | 0.04 | 0.11 | 0.85 | 1.02 |
| Constant | | 0.33 | 0.10 | 0.00 | 0.18 | 0.61 |

Pseudo R-squared: 0.12, Hosmer-Lemeshow chi-square 10.08, p=0.26

1. Socioeconomic status defined by IMD quintile:

|  | | *Odds Ratio* | *Std. Err.* | *P* | *95% Conf. Interval* | |
| --- | --- | --- | --- | --- | --- | --- |
| Availability  (Ref: Equal) | Healthier | 1.53 | 0.44 | 0.14 | 0.87 | 2.67 |
|  | Less healthy | 0.15 | 0.06 | 0.00 | 0.06 | 0.33 |
| IMD quintile (Ref: Quintile 1: Least deprived) | Quintile 2 | 0.94 | 0.28 | 0.83 | 0.53 | 1.67 |
|  | Quintile 3 | 0.51 | 0.16 | 0.03 | 0.28 | 0.93 |
|  | Quintile 4 | 0.93 | 0.27 | 0.79 | 0.53 | 1.63 |
|  | Quintile 5 | 0.62 | 0.19 | 0.13 | 0.34 | 1.14 |
| Healthier availability * Quintile 2 | | 1.22 | 0.52 | 0.64 | 0.53 | 2.81 |
| Healthier availability * Quintile 3 | | 2.74 | 1.19 | 0.02 | 1.17 | 6.41 |
| Healthier availability * Quintile 4 | | 0.73 | 0.30 | 0.43 | 0.33 | 1.61 |
| Healthier availability * Quintile 5 | | 1.87 | 0.78 | 0.14 | 0.82 | 4.25 |
| Less healthy availability * Quintile 2 | | 1.88 | 1.01 | 0.24 | 0.65 | 5.40 |
| Less healthy availability * Quintile 3 | | 2.13 | 1.22 | 0.19 | 0.70 | 6.54 |
| Less healthy availability * Quintile 4 | | 1.08 | 0.65 | 0.90 | 0.33 | 3.50 |
| Less healthy availability * Quintile 5 | | 1.92 | 1.13 | 0.27 | 0.61 | 6.09 |
| High cognitive load | | 1.17 | 0.14 | 0.20 | 0.92 | 1.48 |
| Age | | 1.01 | 0.00 | 0.22 | 1.00 | 1.01 |
| Female | | 1.24 | 0.16 | 0.10 | 0.96 | 1.59 |
| Hunger | | 0.94 | 0.04 | 0.20 | 0.86 | 1.03 |
| Constant | | 0.49 | 0.16 | 0.03 | 0.26 | 0.93 |

Pseudo R-squared: 0.13, Hosmer-Lemeshow chi-square 4.53, p=0.81

**Table S5**. Regression coefficients predicting (i) enjoyment of healthier snack options, (ii) enjoyment of less healthy snack options and (iii) SUPPS-P total scores from socioeconomic status, controlling for availability condition, cognitive load condition, gender, age and hunger

| Outcome | Socioeconomic group | | *Coefficient* | *Std. Err.* | *P* | *95% Conf. Interval* | |
| --- | --- | --- | --- | --- | --- | --- | --- |
| Enjoyment: Healthier snacks | Social class  (Ref: A&B) | C1&C2 | 0.08 | 0.06 | 0.22 | -0.05 | 0.20 |
|  |  | D&E | -0.03 | 0.06 | 0.70 | -0.15 | 0.10 |
|  | Highest educational qualification  (Ref: 1-4 GCSEs) | 5+ GCSEs | -0.13 | 0.10 | 0.20 | -0.33 | 0.07 |
|  |  | 2+ A Levels | -0.29 | 0.12 | 0.01 | -0.52 | -0.06 |
|  |  | Degree or higher | -0.09 | 0.09 | 0.33 | -0.26 | 0.09 |
|  | Income group  (Ref: Up to £17,499) | £17,500-£29,999 | 0.07 | 0.07 | 0.35 | -0.08 | 0.21 |
|  |  | £30,000-£49,999 | 0.06 | 0.07 | 0.40 | -0.08 | 0.20 |
|  |  | £50,000+ | 0.10 | 0.08 | 0.20 | -0.05 | 0.26 |
|  | IMD quintile  (Ref: Quintile 1: Least deprived) | Quintile 2 | 0.04 | 0.09 | 0.61 | -0.13 | 0.21 |
|  |  | Quintile 3 | -0.02 | 0.09 | 0.81 | -0.19 | 0.15 |
|  |  | Quintile 4 | 0.11 | 0.09 | 0.20 | -0.06 | 0.28 |
|  |  | Quintile 5 | 0.12 | 0.09 | 0.17 | -0.05 | 0.29 |
| Enjoyment: Less healthy snacks | Social class  (Ref: A&B) | C1&C2 | 0.14 | 0.07 | 0.04 | 0.01 | 0.26 |
|  |  | D&E | 0.09 | 0.07 | 0.17 | -0.04 | 0.22 |
|  | Highest educational qualification  (Ref: 1-4 GCSEs) | 5+ GCSEs | -0.10 | 0.11 | 0.34 | -0.31 | 0.11 |
|  |  | 2+ A Levels | -0.26 | 0.12 | 0.03 | -0.50 | -0.03 |
|  |  | Degree or higher | -0.19 | 0.09 | 0.03 | -0.37 | -0.02 |
|  | Income group  (Ref: Up to £17,499) | £17,500-£29,999 | 0.09 | 0.08 | 0.24 | -0.06 | 0.24 |
|  |  | £30,000-£49,999 | 0.08 | 0.07 | 0.28 | -0.06 | 0.22 |
|  |  | £50,000+ | 0.03 | 0.08 | 0.75 | -0.13 | 0.19 |
|  | IMD quintile  (Ref: Quintile 1: Least deprived) | Quintile 2 | -0.10 | 0.09 | 0.26 | -0.27 | 0.07 |
|  |  | Quintile 3 | -0.03 | 0.09 | 0.72 | -0.20 | 0.14 |
|  |  | Quintile 4 | 0.15 | 0.09 | 0.08 | -0.02 | 0.33 |
|  |  | Quintile 5 | 0.09 | 0.09 | 0.29 | -0.08 | 0.27 |
| SUPPS-P score | Social class  (Ref: A&B) | C1&C2 | -0.17 | 0.57 | 0.77 | -1.28 | 0.94 |
|  |  | D&E | 0.76 | 0.57 | 0.18 | -0.36 | 1.87 |
|  | Highest educational qualification  (Ref: 1-4 GCSEs) | 5+ GCSEs | -1.96 | 0.92 | 0.03 | -3.77 | -0.15 |
|  |  | 2+ A Levels | -1.76 | 1.05 | 0.09 | -3.83 | 0.30 |
|  |  | Degree or higher | -1.32 | 0.79 | 0.10 | -2.88 | 0.24 |
|  | Income group  (Ref: Up to £17,499) | £17,500-£29,999 | -0.09 | 0.65 | 0.89 | -1.37 | 1.18 |
|  |  | £30,000-£49,999 | -0.50 | 0.64 | 0.43 | -1.75 | 0.74 |
|  |  | £50,000+ | -0.97 | 0.70 | 0.17 | -2.35 | 0.40 |
|  | IMD quintile  (Ref: Quintile 1: Least deprived) | Quintile 2 | 0.40 | 0.75 | 0.59 | -1.08 | 1.88 |
|  |  | Quintile 3 | -0.53 | 0.75 | 0.48 | -2.00 | 0.94 |
|  |  | Quintile 4 | 0.56 | 0.75 | 0.46 | -0.93 | 2.04 |
|  |  | Quintile 5 | 1.08 | 0.76 | 0.16 | -0.42 | 2.58 |

**Table S6**. Results of logistic regression predicting healthier food choice from availability conditions, with enjoyment ratings and impulsivity (SUPPS-P) as predictors.

|  | | *Odds Ratio* | *Std. Err.* | *P* | *95% Conf. Interval* | |
| --- | --- | --- | --- | --- | --- | --- |
| Availability  (Ref: Equal) | Healthier | 2.26 | 0.31 | 0.00 | 1.72 | 2.97 |
|  | Less healthy | 0.21 | 0.04 | 0.00 | 0.15 | 0.29 |
| High cognitive load | | 1.19 | 0.15 | 0.16 | 0.93 | 1.52 |
| Enjoyment ratings: Healthier food | | 2.17 | 0.18 | 0.00 | 1.84 | 2.57 |
| Enjoyment ratings: Less healthy food | | 0.41 | 0.03 | 0.00 | 0.35 | 0.49 |
| SUPPS-P total score | | 1.02 | 0.01 | 0.01 | 1.00 | 1.03 |
| Social class  (Ref: A&B) | C1&C2 | 0.96 | 0.15 | 0.79 | 0.71 | 1.29 |
|  | D&E | 0.83 | 0.13 | 0.23 | 0.62 | 1.12 |
| Age | | 1.00 | 0.00 | 0.40 | 0.99 | 1.01 |
| Female | | 1.10 | 0.14 | 0.48 | 0.85 | 1.42 |
| Hunger | | 0.97 | 0.05 | 0.51 | 0.88 | 1.06 |
| Constant | | 0.32 | 0.15 | 0.01 | 0.13 | 0.78 |

Pseudo R-squared: 0.19, Hosmer-Lemeshow chi-square 6.45, p=0.60
